# Role of diffusion-weighted imaging in the diagnosis of pituitary region tumors

**DOI:** 10.1007/s00234-024-03467-z

**Published:** 2024-09-28

**Authors:** Adrian Korbecki, Justyna Wagel, Anna Zacharzewska-Gondek, Maja Gewald, Justyna Korbecka, Michał Sobański, Arkadiusz Kacała, Agata Zdanowicz-Ratajczyk, Maciej Kaczorowski, Agnieszka Hałoń, Grzegorz Trybek, Stylianos Kapetanakis, Joanna Bladowska

**Affiliations:** 1 Department of General Radiology, Interventional Radiology and Neuroradiology, University Clinical Hospital, Borowska 213, 50–556, Wroclaw, Poland; 2 Wroclaw Medical University, Wroclaw, Poland; 3 Department of Neurology, Wroclaw Medical University, Wroclaw, Poland; 4 Department of General Radiology, Interventional Radiology and Neuroradiology, Wroclaw Medical University, Wroclaw, Poland; 5 Department of Clinical and Experimental Pathology, Wroclaw Medical University, Wroclaw, Poland; 6 Department of Oral Surgery, Pomeranian Medical University in Szczecin, Szczecin, Poland; 7 Maxillofacial Surgery Clinic, Wroclaw 4th Military Clinical Hospital, Wroclaw, Poland; 8 Spine Department and Deformities, Interbalkan European Medical Center, Thessaloniki, Greece; 9 Department of Preclinical Sciences, Pharmacology and Medical Diagnostics, Faculty of Medicine, Wroclaw University of Science and Technology, Wroclaw, Poland; 10 Department of Radiology, Wroclaw 4th Military Clinical Hospital, Wroclaw, Poland

**Keywords:** Pituitary tumors, Pituitary adenoma, Meningioma, Diffusion weighted MRI, Magnetic resonance imaging

## Abstract

**Purpose:**

This study aimed to assess the role of Diffusion-Weighted Imaging (DWI) in routine pituitary Magnetic Resonance Imaging (MRI) protocols for distinguishing sellar and parasellar tumors, addressing the lack of clear guidelines in contemporary literature.

**Methods:**

A retrospective analysis of 242 pituitary MRI scans with DWI sequences was conducted in a single-center study using a 1.5 T scanner and standard DWI sequence parameters. Measurements of both absolute and relative mean apparent diffusion coefficient (ADC) values, along with minimal ADC values within tumors, were performed. The adopted region of interest (ROI) based method used for these measurements was validated.

**Results:**

Invasive pituitary adenomas exhibited significantly lower min ADC and min rADC than meningiomas, with optimal cut-off points of 0.64 (sensitivity 73%, specificity 82%) and 0.78 (sensitivity 73%, specificity 89%), respectively. Post-hemorrhagic pituitary adenomas demonstrated lower ADC values than adamantinomatous craniopharyngiomas, with an AUC of 0.893 for min rADC = 1.07, and Rathke’s Cleft Cysts with mucous content, AUC 0.8 for min rADC = 1.01. Specific differentiation with high sensitivity and specificity based on diffusion parameters was observed for these tumor groups. Cystic pituitary non-functional adenomas obtained significantly lower ADC values compared to the adamantinomatous type of craniopharyngiomas and serous Rathke’s Cleft Cysts (AUC up to 0.942).

**Conclusions:**

The study concludes that integrating DWI into routine pituitary MRI protocols enhances diagnostic accuracy in distinguishing sellar and parasellar tumors. The short scan time of one minute makes DWI a valuable and precise tool, supporting its recommendation as a standard component of pituitary MRI examinations.

## Introduction

Pituitary adenomas and other tumors, such as meningiomas, craniopharyngiomas, and Rathke’s cleft cysts (RCCs) are found in the sellar and parasellar region. The variety of pathologies and the pituitary gland's crucial functions necessitate precise imaging for effective treatment.

Imaging of the pituitary region is a complex issue [1]. Magnetic resonance imaging (MRI) is currently the method of choice for evaluating the pituitary gland [2, 3]. However, the capabilities of conventional MRI sequences on their own are limited, as different pituitary region lesions may present similar morphologies [4].

Advancement of MRI techniques used in diagnostic pituitary lesions, such as 3D volumetric analysis of pituitary volume, diffusion-weighted imaging (DWI), perfusion-weighted imaging (PWI) and magnetic resonance spectroscopy (MRS) allows for a better evaluation of the sellar and parasellar region and leads to proper further management [5, 6]. Research has demonstrated that DWI and apparent diffusion coefficient (ADC) values correlate with cellularity and intraoperative consistency [7–10], diagnosis and degree of aggressiveness and tumor atypia [11, 12], enabling early detection of acute pituitary apoplexy [13]. However, there are few scientific publications that raise the subject of DWI usefulness in the differential diagnosis of sellar and parasellar lesions [14–18]. The results of these are often ambiguous and based on limited patient groups.

The purpose of our study was to evaluate the usefulness of DWI with ADC values measurements in the differential diagnosis of sellar and parasellar tumors. To our knowledge, this will be the first large-scale study which includes such a wide group of examined and histopathologically confirmed tumors.

Moreover, such an analysis will provide answers if DWI should be included in routine pituitary MRI protocols, taking into account both the time of scanning, with regard to the cost effectiveness of patient re-examination, and the role in accurate diagnosis, leading to proper patient management with a special emphasis on avoiding surgical procedures.

## Materials and methods

### Materials

Study included a total number of 242 patients with sellar and parasellar tumors, who underwent an MRI examination with the DWI sequence in the protocol, conducted from October 2007 to April 2023 in a single medical center. Histopathological examination confirmed the diagnosis of 201 out of 242 tumors (83%). The remaining 41 lesions were identified based on typical features on imaging studies, assessed by two experienced neuroradiologists, or on a positive response to bromocriptine pharmacological treatment. The total number 242 examinations were divided into 13 groups, with one group encompassing rare sellar pathologies with fewer than ten cases (Table 1).

**Table 1:** Classification of sellar and parasellar pathologies in the study group (*N* = 242)

Tumor type	*n* (%)	Patient age, years M (SD)
Hormonaly inactive adenomas		
Non-functional solid pituitary adenomas	**93 (38.4)**	63.66 (11.63)
Non-functional cystic pituitary adenomas	**12 (5.0)**	44.58 (23.77)
Hormone-secreting adenomas		
Solid Prolactinomas	**21 (8.7)**	58.67 (15.73)
Cystic prolactinomas	**7 (2.9)**	52.14 (21.54)
Growth hormone secreting adenomas	**4 (1.7)**	54.50 (11.47)
ACTH-secreting adenomas	**2 (0.8)**	66.50 (2.12)
Meningiomas	**38 (15.7)**	48.81 (17.35)
Craniopharyngiomas		
Adamantinomatous craniopharyngiomas	**19 (7.9)**	63.29 (11.38)
Papillary craniopharyngiomas	**3 (1.2)**	51.33 (9.07)
Rathke’s cleft cysts		
Rathke’s cleft cysts (serous)	**10 (4.1)**	57.30 (14.09)
Rathke’s cleft cysts (mucinous)	**10 (4.1)**	41.30 (26.54)
Not specifically classified adenomas *	**3 (1.2)**	48.00 (14.11)
Hemorrhagic pituitary adenomas **	**19 (7.9)**	57.63 (18.03)
Invasive adenoma **	**11 (4.5)**	61.45 (15.84)
**Rare pituitary region pathologies**	**20 (8.3)**	‒

^*^excluded from main groups due to lack of precise information

^**^additionally selected from the main study groups for statistical analysis

The exclusion criteria encompassed tumors smaller than 1.0 cm in both the anterior–posterior and cranio-caudal dimensions (approximately twice the slice thickness), as well as significant artifacts resulting from calcifications within the tumor.

The most numerous groups included non-functional solid adenomas (*n* = 93), meningiomas (*n* = 38), prolactinomas (*n* = 21), adamantinomatous craniopharyngiomas (*n* = 19), and non-functional cystic pituitary adenomas (*n* = 12). Rathke's cleft cysts were divided into two subgroups based on their content – the serous type (*n* = 10), when the content exhibited a signal similar to cerebrospinal fluid in T1- and T2-weighted sequences, and the mucinous type (*n* = 10) characterized by high signal intensity in T1-weighted images and low signal intensity on T2-weighted images. Rare pituitary region pathologies were combined into one group listed in Table 1, as the small number of cases prevented further detailed analysis.

Adenomas were classified as cystic, if this type of morphology exceeded 50% of the tumor.

Not specifically classified adenomas (*n* = 3) are the lesions excluded from the main groups due to the presence of severe hemorrhage comprising the entire area of the tumor.

From all of the previously mentioned tumors two additional groups were selected – hemorrhagic pituitary adenomas and invasive adenomas. The first of these comprises every adenoma, in which features of hemorrhage were present. The second one consists of tumors that have infiltrated the sella turcica, cavernous sinus, sphenoid sinus or dura mater, according to Hansen et al. [19].

### Imaging data

The MRI examinations were performed on a GE Signa Hdx 1.5 T scanner with a 16-channel coil. Standard sequences for pituitary MRI examination included multiplanar T1-,T2-, and axial 3D T1-weighted after intravenous administration of gadolinium contrast. Standard MRI sequences were used to assess the dimensions of the lesion, morphology and the presence of the hemorrhage.

DWI data were acquired with axially-oriented single-shot spin-echo echo-planar imaging with a b-value of 0 mm^2^/s and 1 000 mm^2^/s. The parameters were identical to a typical brain MRI protocol: repetition time (TR) = 10,000 ms, echo time (TE) = 82.7 ms, field of view (FOV) = 24 cm, matrix size = 128 × 128, slice thickness = 5 mm, slice spacing = 1 mm, number of excitations (NEX) = 5, total scan time = 1:18.

### Imaging analysis

The DWI sequence was post-processed on a dedicated GE Medical Systems ADW 4.6 diagnostic workstation with Functool software provided by the manufacturer.

Absolute ADC values were measured as mean values (mean ADC) and minimum values (min ADC). The mean ADC value is the arithmetic average from ADC measurements obtained by outlining the whole lesion with an region of interest (ROI) on all axial slices that constrained the pathology. The min ADC value is the lowest value measured with a ROI of approximately 30 mm^2^ selected from all cross-sections of the lesion.

**Fig. 1:**
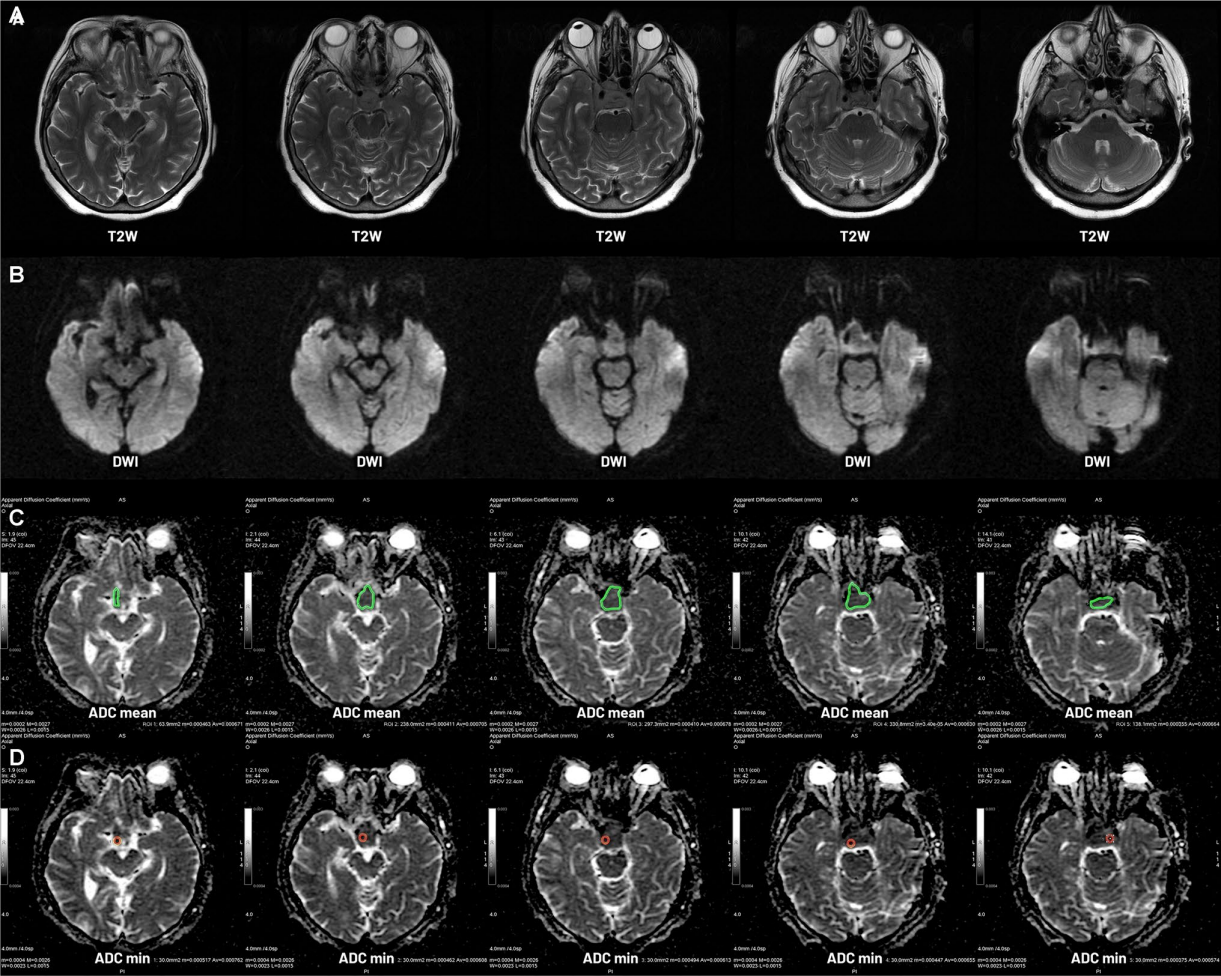
Axial T2-weighted images present a sellar and suprasellar meningioma (A), which is also visible on DWI images (B). The mean ADC measurements are obtained by outlining the whole lesion with a region of interest (ROI) on all axial slices of the ADC map that constrained the pathology (C). The min ADC value is the lowest value measured with an ROI of approximately 30 mm.^2^ selected from all cross-sections of the lesion (D)

Relative ADC values were acquired by comparing the absolute values to normal-appearing white matter. An average of up to five measurements performed within the ipsilateral temporal lobe with a round ROI of approximately 60 mm^2^ was calculated. Next, the measurements from the first step of analysis (mean ADC and min ADC) were divided by the ADC value of the normal-appearing white matter to obtain relative values (mean rADC and min rADC). This approach ensures greater repeatability and generalized applications of our study (Fig. 1).

In the conventional MRI images, all the lesions were measured in the largest anteroposterior (AP), transversal (TR) and craniocaudal (CC) dimensions.

### Statistical analysis

Statistical analysis was performed in R software (version 4.1.2]. Absolute and relative frequencies were used to describe nominal parameters. Numeric variables were described with basic descriptive statistics. At first, ROC (receiver operating characteristics) analysis was run to evaluate the ability of DWI parameters to predict each tumor type with at least 10 observations vs all other tumor types. Secondly, deep-dive analysis was prepared, in which the ROC algorithm was used for selected tumor types to evaluate the ability of DWI parameters to predict the selected tumor types vs other tumor types. The basic condition on which to include tumors as “other tumor types” was at least 10 observations. The Youden method was employed to evaluate the best thresholds. Statistical tests assumed α < 0.05.

## Results

### Study sample

From October 2007 to April 2023, a total of 242 patients with sellar and parasellar tumors underwent MRI examination including a DWI sequence. Women represented 54.1% (131) of the group, men represented 45.9% (111). The mean age and standard deviation (SD) were 56.58 ± 18.03 years.

### Initial analysis – one vs all others

Preliminary evaluation of qualitative features between one tumor group and all other tumors combined, within the groups containing at least 10 observations, confirmed several statistically significant changes. Detailed results are summarized in Table 2 (Table 2).

**Table 2:** ROC analysis outcomes – AUC comparison for sellar and parasellar pathologies (vs all others)

**Variable**	Non-functional solid pituitary adenomas	Non-functional cystic pituitary adenomas	Solid Prolactinomas	Hemorrhagic pituitary adenomas	Invasive adenoma	Meningiomas	Adamantinomatous craniopharyngiomas	Rathke’s cleft cysts (serous)	Rathke’s cleft cysts (mucinous)
TR	‒	‒	0.707	‒	0.830	‒	‒	0.825	0.853
AP	‒	0.678	0.649	‒	0.798	0.718	0.662	0.860	0.929
CC	‒	0.678	0.672	‒	0.850	‒	‒	0.793	0.839
Volume	‒	0.667	‒	‒	‒	‒	‒	0.847	0.906
ADC mean	0.726	0.746	0.619	‒	0.618	0.603	0.888	0.954	‒
rADC mean	0.721	0.746	0.608	‒	0.636	0.620	0.885	0.945	‒
ADC minimum	0.755	‒	0.660	0.693	0.765	‒	0.816	0.971	‒
rADC minimum	0.750	‒	0.660	0.700	0.782	0.531	0.808	0.965	‒

### Profound analysis – one vs one

Based on prior ROC analysis, selected groups were compared pairwise to determine the most appropriate thresholds for clinical applications.

As expected, the comparison between solid and cystic sellar and parasellar tumors demonstrated high AUC values, sensitivity, and specificity, often reaching 100%. The same highly accurate correlations were found for distinguishing between serous and mucinous RCCs. These results are listed in Table 3 and 4.

**Table 3:** Cut-off points and AUC for predicting sellar and parasellar pathologies, based on ROC analysis

Tumor type	n	AP, mm	TR, mm	CC, mm	Volume, mm^3^	ADC mean × 10^-3 mm2/sec / rADC mean	ADC min × 10^-3 mm2/sec / rADC min		
		M (SD)	M (SD)	M (SD)	M (SD)	M (SD)	Range	M (SD)	Range
Hormonaly inactive adenomas									
Non-functional solid pituitary adenomas	93	2.08 (0.88)	2.27 (0.86)	2.40 (1.29)	8.99 (12.36)	0.93 (0.29)/ 1.18 (0.37)	0.40–1.86/ 0.52–2.49	0.70 (0.28)/ 0.89 (0.35)	0.28–1.79/ 0.31–2.42
Non-functional cystic pituitary adenomas	19	2.85 (1.44)	2.81 (1.35)	2.97 (2.02)	24.67 (50.29)	2.16 (0.30)/ 2.62 (0.38)	1.72–2.95/ 2.07–3.51	1.46 (0.38)/ 1.78 (0.49)	0.83–2.06/ 0.97–2.75
Hormone-secreting adenomas									
Solid Prolactinomas	12	1.61 (0.66)	1.93 (0.69)	1.63 (0.88)	3.69 (4.13)	1.74 (0.54)/ 2.15 (0.68)	0.74–2.39/ 0.96–2.97	1.32 (0.58)/ 1.63 (0.68)	0.64–2.36/ 0.83–2.59
Cystic prolactinomas	7	1.54 (0.50)	1.80 (0.80)	1.57 (0.79)	2.89 (2.73)	2.36 (0.33)/ 2.88 (0.37)	1.78–2.74/ 2.37–3.41	1.92 (0.44)/ 2.36 (0.57)	1.20–2.54/ 1.38–3.07
Growth hormone secreting adenomas	4	1.52 (0.34)	1.50 (0.37)	1.60 (0.48)	2.15 (1.65)	0.75 (0.21)/ 0.97 (0.23)	0.61–1.06/ 0.83–1.31	0.62 (0.23)/ 0.80 (0.28)	0.35–0.92/ 0.46–1.14
ACTH-secreting adenomas	2	1.55 (0.92)	2.10 (1.56)	2.10 (1.41)	5.97 (7.71)	1.24 (0.41)/ 1.52 (0.46)	0.95–1.53/ 1.20–1.84	1.10 (0.51)/ 1.35 (0.58)	0.74–1.46/ 0.94–1.76
Meningiomas	21	2.80 (1.26)	3.27 (1.44)	3.11 (1.58)	21.81 (21.12)	0.96 (0.31)/ 1.21 (0.34)	0.50–1.69/ 0.65–2.13	0.71 (0.30)/ 0.88 (0.33)	0.35–1.54/ 0.45–1.68
Craniopharyngiomas									
Adamantinomatous craniopharyngiomas	38	2.91 (1.14)	2.69 (1.32)	2.35 (1.17)	14.37 (20.14)	0.95 (0.18)/ 1.18 (0.21)	0.70–1.38/ 0.79–1.72	0.80 (0.20)/ 1.00 (0.23)	0.28–1.26/ 0.36–1.48
Papillary craniopharyngiomas	3	2.67 (0.29)	2.73 (0.51)	2.80 (1.28)	11.69 (8.77)	1.81 (0.41)/ 2.39 (0.37)	1.56–2.29/ 2.14–2.82	1.23 (0.25)/ 1.63 (0.21)	1.06–1.51/ 1.49–1.86
Rathke’s cleft cysts									
Rathke’s cleft cysts (serous)	10	1.14 (0.53)	1.31 (0.65)	1.29 (0.67)	1.55 (2.58)	2.62 (0.44)/ 3.16 (0.53)	1.70–3.33/ 2.03–3.97	2.45 (0.45)/ 2.96 (0.57)	1.51–3.06/ 1.80–3.97
Rathke’s cleft cysts (mucinous)	10	0.91 (0.34)	1.24 (0.37)	1.12 (0.46)	0.81 (0.92)	1.18 (0.22)/ 1.44 (0.27)	0.84–1.51/ 1.01–1.82	1.11 (0.27)/ 1.35 (0.32)	0.54–1.51/ 0.68–1.82
** Hemorrhagic pituitary adenomas**	19	2.52 (0.94)	2.82 (1.01)	2.92 (1.08)	14.12 (13.00)	1.04 (0.57)/ 1.29 (0.67)	0.22–2.32/ 0.27–2.97	0.71 (0.43)/ 0.88 (0.51)	0.13–1.78/ 0.16–2.01
** Invasive adenoma**	11	3.54 (1.30)	3.64 (1.00)	4.01 (1.44)	31.65 (26.98)	0.92 (0.17)/ 1.14 (0.21)	0.66–1.22/ 0.82–1.47	0.58 (0.18)/ 0.72 (0.23)	0.28–0.91/ 0.31–1.17

*M*—mean, *SD*—standard deviation

^*^ No SD due to *n* = 1 counts

**Table 4:** Descriptive statistics for sellar and parasellar pathologies

Comparison	ADC mean		rADC mean		ADC min		rADC min	
	Cut-off	AUC (95% CI)	Cut-off	AUC (95% CI)	Cut-off	AUC (95% CI)	Cut-off	AUC (95% CI)
Comparisons of pathologies with markedly different morphology on conventional MRI images								
Meningiomas vs								
Adamantinomatous craniopharyngiomas*	1.55	1.000 (1.000;1.000)	1.89	1.000 (1.000;1.000)	1.32	0.941 (0.881;1.000)	1.36	0.931 (0.863;0.998)
Rathke’s cleft cysts (serous)*	1.54	1.000 (1.000;1.000)	1.88	1.000 (1.000;1.000)	1.39	1.000 (1.000;1.000)	1.64	1.000 (1.000;1.000)
Non-functional cystic pituitary adenomas*	1.07	0.895 (0.744;1.000)	1.62	0.893 (0.741;1.000)	‒	‒	‒	
Rathke’s cleft cysts (mucinous)*	‒	‒	‒	‒	1.03	0.826 (0.635;1.000)	1.32	0.816 (0.626;1.000)
Solid Prolactinomas vs								
Adamantinomatous craniopharyngiomas*	1.71	1.000 (1.000;1.000)	1.91	0.997 (0.991;1.000)	0.82	0.932 (0.859;1.000)	1.12	0.935 (0.866;1.000)
Rathke’s cleft cysts (serous)*	1.69	1.000 (1.000;1.000)	1.89	0.995 (0.982;1.000)	1.31	0.995 (0.982;1.000)	1.74	1.000 (1.000;1.000)
Non-functional cystic pituitary adenomas*	1.24	0.885 (0.749;1.000)	1.35	0.893 (0.760;1.000)	1.28	0.829 (0.686;0.973)	1.71	0.825 (0.675;0.976)
Rathke’s cleft cysts (mucinous)*	1.16	0.745 (0.561;0.929)	‒	‒	1.03	0.850 (0.695;1.000)	1.01	0.838 (0.677;0.999)
Non-functional solid pituitary adenomas vs								
Rathke’s cleft cysts (serous)*	1.67	0.997 (0.989;1.000)	2.02	0.996 (0.986;1.000)	1.48	0.997 (0.989;1.000)	1.80	0.997 (0.989;1.000)
Adamantinomatous craniopharyngiomas*	1.68	0.993 (0.984;1.000)	2.06	0.989 (0.975;1.000)	0.81	0.949 (0.910;0.988)	1.13	0.939 (0.894;0.985)
Non-functional cystic pituitary adenomas*	1.24	0.890 (0.770;1.000)	1.36	0.889 (0.771;1.000)	0.75	0.853 (0.748;0.959)	0.83	0.842 (0.731;0.952)
Rathke’s cleft cysts (mucinous)*	1.03	0.790 (0.674;0.906)	1.39	0.763 (0.632;0.895)	0.84	0.859 (0.723;0.994)	1.18	0.848 (0.709;0.988)
Invasive adenoma vs								
Adamantinomatous craniopharyngiomas*	1.47	1.000 (1.000;1.000)	1.77	1.000 (1.000;1.000)	0.79	0.990 (0.968;1.000)	0.95	0.986 (0.954;1.000)
Rathke’s cleft cysts (serous)*	1.46	1.000 (1.000;1.000)	1.75	1.000 (1.000;1.000)	1.21	1.000 (1.000;1.000)	1.49	1.000 (1.000;1.000)
Non-functional cystic pituitary adenomas*	1.24	0.924 (0.800;1.000)	1.47	0.932 (0.810;1.000)	0.75	0.928 (0.828;1.000)	0.80	0.917 (0.806;1.000)
Rathke’s cleft cysts (mucinous)*	1.15	0.823 (0.633;1.000)	1.37	0.809 (0.611;1.000)	0.80	0.936 (0.823;1.000)	0.97	0.936 (0.823;1.000)
Comparisons of pathologies with overlapping morphology on conventional MRI images								
Invasive adenoma vs Meningiomas*	‒	‒	‒	‒	0.64	0.817 (0.662;0.972)	0.78	0.823 (0.668;0.978)
Rathke’s cleft cysts (mucinous)* vs Hemorrhagic pituitary adenomas	‒	‒	‒	‒	0.83	0.800 (0.623;0.977)	1.01	0.795 (0.614;0.976)
Adamantinomatous craniopharyngiomas* vs Hemorrhagic pituitary adenomas	1.53	0.906 (0.788;1.000)	1.92	0.917 (0.812;1.000)	0.82	0.898 (0.788;1.000)	1.07	0.893 (0.781;1.000)
Non-functional cystic pituitary adenomas vs Adamantinomatous craniopharyngiomas*	1.75	0.700 (0.485;0.914)	2.12	0.706 (0.493;0.919)	‒	‒	‒	‒
Non-functional cystic pituitary adenomas vs Rathke’s cleft cysts (serous)*	2.32	0.925 (0.802;1.000)	2.93	0.892 (0.743;1.000)	2.10	0.938 (0.839;1.000)	2.48	0.942 (0.837;1.000)
Adamantinomatous craniopharyngiomas vs Rathke’s cleft cysts (serous)*	2.32	0.837 (0.636;1.000)	2.94	0.811 (0.604;1.000)	2.12	0.958 (0.872;1.000)	2.47	0.942 (0.836;1.000)
Adamantinomatous craniopharyngiomas* vs Rathke’s cleft cysts (mucinous)	1.62	1.000 (1.000;1.000)	1.94	1.000 (1.000;1.000)	1.36	0.763 (0.583;0.943)	1.62	0.758 (0.580;0.935)
Rathke’s cleft cysts (mucinous) vs Rathke’s cleft cysts (serous)*	1.60	1.000 (1.000;1.000)	1.93	1.000 (1.000;1.000)	1.85	0.995 (0.981;1.000)	2.19	0.990 (0.962;1.000)

AUC—area under curve, CI—confidence interval. Cut-offs for ADC and rADC presented as × 10^-3 mm^2^/sec. All presented outcomes were significant (*p* < 0.05)

^*^Type predicted when above cut-off point

Invasive pituitary adenomas showed ADC min = 0.58 ± 0.18 × 10^−3^mm^2^/s, rADC min = 0.72 ± 0.23, whereas meningiomas displayed higher values, both for ADC min and rADC min, specifically 0.80 ± 0.20 × 10^−3^mm^2^/s and 1.00 ± 0.23. The optimal cut-off points were as follows:

‒ For ADC min > 0.64 × 10^−3^mm^2^/s, differentiation between meningiomas and invasive pituitary adenomas achieved a sensitivity of 73% and specificity of 82%.
‒ For rADC min > 0.78, differentiation between meningiomas and invasive pituitary adenomas achieved a sensitivity of 73% and specificity of 89%.

Hemorrhagic pituitary adenomas yielded significantly lower ADC values (ADC min = 0.71 ± 0.43 × 10^−3^mm^2^/s and rADC min = 0.88 ± 0.51) compared to adamantinomatous craniopharyngiomas (ADC min = 1.46 ± 0.38 × 10^−3^mm^2^/s and rADC min = 1.78 ± 0.49) and mucinous Rathke’s cleft cyst (ADC min = 1.11 ± 0.27 × 10^−3^mm^2^/s and rADC min = 1.35 ± 0.32).

The optimal cut-off points of ADC mean > 1.53 × 10^−3^mm^2^/s, rADC mean > 1.92, ADC min > 0.82 × 10^−3^mm^2^/s and rADC min > 1.07 enabled differentiation of adamantinomatous craniopharyngiomas from hemorrhagic pituitary adenomas with sensitivities exceeding 95% and specificities of 84%, 84%, 79% and 79%, respectively.

Thresholds of ADC min > 0.83 × 10^−3^mm^2^/s and rADC min > 1.01 effectively differentiated mucinous RCCs from hemorrhagic pituitary adenomas with a sensitivity of 90% and specificity of 79%.

In the case of comparing cystic pituitary adenomas with adamantinomatous craniopharyngiomas and serous Rathke’s cleft cyst, cystic pituitary adenomas exhibited significantly lower ADC values (Tables 3,4). The following cut-off points with the highest accuracy were identified:

– ADC mean > 1.75 × 10^−3^mm^2^/s and rADC mean > 2.12 differentiated adamantinomatous craniopharyngiomas from cystic pituitary adenomas with a sensitivity of 50% and specificity of 95%.
– ADC mean > 2.32 × 10^−3^mm^2^/s, ADC min > 2.10 × 10^−3^mm^2^/s and rADC min > 2.48 enabled the differentiation of serous RCCs from cystic pituitary adenomas with sensitivity of 90% and specificity of 92%.
– rADC mean > 2.93 effectively differentiated serous RCCs from cystic pituitary adenomas with a sensitivity of 80% and specificity of 92%.
- ADC values for adamantinomatous craniopharyngiomas were higher than mucinous and lower than serous RCCs (Tables 3,4):
– ADC mean > 1.62 × 10^−3^mm^2^/s, rADC mean > 1.94 effectively differentiated adamantinomatous craniopharyngiomas from mucinous RCCs with sensitivities and specificities of 100%.
– ADC mean > 2.32 × 10^−3^mm^2^/s, rADC mean > 2.94, ADC min > 2.12 × 10^−3^mm^2^/s and rADC min > 2.47 effectively distinguished serous RCCs from adamantinomatous craniopharyngiomas with sensitivities of 84%, 84%, 100%, 95% and specificities of 90%, 80%, 90% and 90%, respectively.

Detailed ADC values are depicted in Table 3.

## Discussion

In our research, we examined a population of 242 patients, with 201 cases confirmed through histopathological examination – the largest cohort of sellar and parasellar tumors analyzed in a single study in contemporary literature. Consequently, we precisely classified pituitary tumors. This method of group separation may be the reason for observed ADC differences compared to other publications, where some groups were discussed collectively.

It is important to emphasize that many tumor pairs achieved high or even perfect areas under the curve values (AUC = 1). These outcomes were observed when comparing cystic to solid pathologies and have limited clinical relevance, as similar information can be obtained from conventional T1-weighted and T2-weighted sequences. These steps in our study were necessary to validate the adopted conventional ROI-based methods as effective and repeatable.

The method of ROI-based ADC measurement within the tumor varies among publications. ADC values were assessed using six or more ROI, which ranged in size from 8 mm^2^ to 77 mm^2^ [12, 15, 20, 21]. ROIs were placed in the central part of the tumor [9, 16, 17], in the solid part of the tumor [7, 15] or in the cystic part [14, 20]. In our study we employed two methods of measurement to obtain more objective results – it included mean ADC and rADC values averaged from outlining the entire tumor on all axial slices (excluding calcified or posthemorrhagic regions), as well as minimal ADC and rADC values chosen from all axial slices. Our study focused on ROI-based methods, a swift and practical solution for everyday routine clinical use, eliminating the need for extracting data and post-processing outside the vendor application.

The last source of differences is the technical aspects of the DWI sequence. In our study, we consistently used axially-oriented single-shot spin-echo echo-planar imaging, as it is a standard sequence that can be widely applied in multiple medical centers [7, 22]. Other studies have utilized different approaches [14, 21] while recent publications have explored the advantages of newer techniques, such as turbo field echo with diffusion sensitized driven equilibrium (DSDE-TFE), which aims to minimize susceptibility artifacts and improve spatial resolution [23, 24].

Invasive adenomas and other solid tumors, such as meningiomas, may exhibit similar imaging features in basic MRI sequences, including skull base and meningeal infiltration [25]. In our study, both of these pathologies yielded comparable ADC results to the majority of previous publications [12, 16, 26, 27]. Furthermore, we consistently acquired significantly higher ADC min and rADC min parameters of meningiomas compared to invasive adenomas. Our results, which are based on well-defined groups, strongly suggest that an rADC min value exceeding 0.78 (with 73% sensitivity and 89% specificity) can effectively differentiate meningiomas from invasive pituitary adenomas. As this threshold is a relative value, we recommend using this method in routine clinical practice.

Simultaneously, we found no significant correlations between solid non-functional and PRL-secreting adenomas. In the current literature, only Mohammad et al. and Yamasaki et al. have investigated the utility of DWI in differential diagnosis, with conflicting results [16, 18]. This disparity could be due to group inhomogeneity and small sample sizes.

**Fig. 2:**
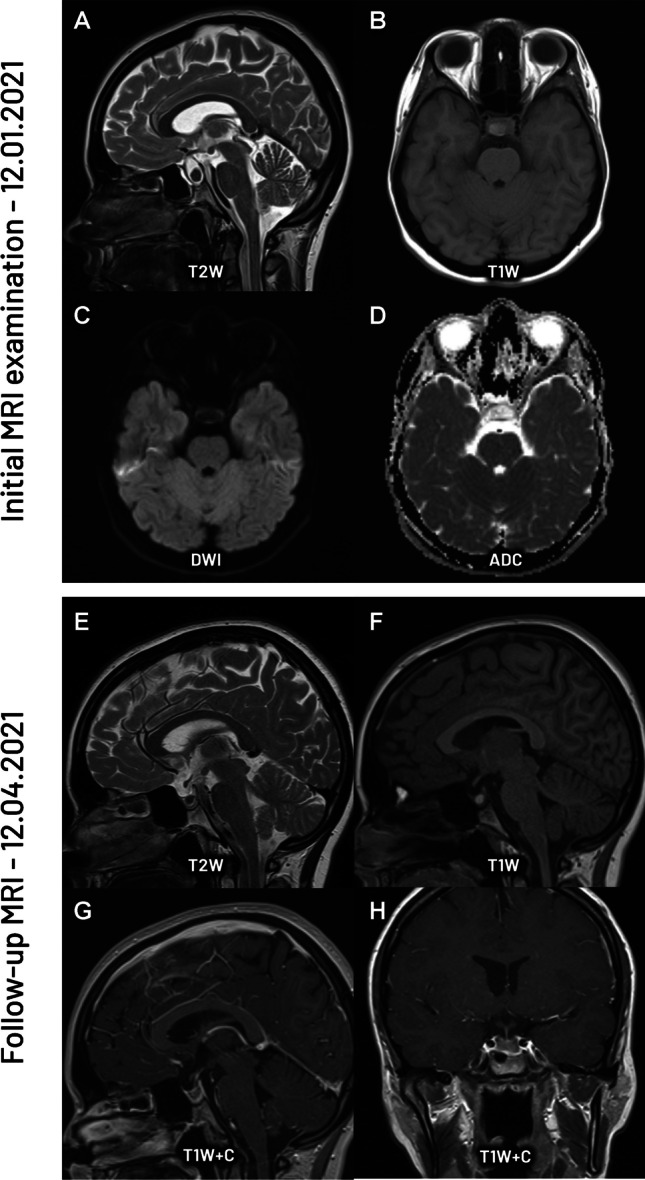
Initial MRI: sagittal T2-weighted image (A), axial T1-weighted image (B), axial DWI (C), ADC map (D). There is a tumor presenting high signal intensity region on T1 that was interpreted as a hemorrhage. On DWI (C), the tumor does not show features of reduced diffusion with high signal on the ADC map (D), indicating no bleeding but suggesting a high protein content. The follow-up MRI performed four months later confirms the initial diagnosis of Rathke’s cleft cyst, with regression of the size of the lesion on sagittal T2-weighted image (E), sagittal T1-weighted image (E), and post-contrast sagittal (G) and coronal (H) T1-weighted images

Hemorrhagic pituitary adenomas, adamantinomatous craniopharyngiomas, and Rathke's cleft cysts may present with similar morphologies and overlapping signals on conventional T1- and T2-weighted sequences usually showing high signal intensity on T1-weighted images [14, 15, 28]. This is caused by the presence of intra- and extracellular methemoglobin during the subacute phase of hemorrhage, while in the other two groups, it results from variations in protein and cholesterol concentrations. Differentiation of the pathologies is crucial to avoid unnecessary operations in case of RCCs, and decide which lesion may be treated conservatively or simply by follow-up examinations, as RCCs tend to show spontaneous regression (Fig. 2).

In our study, we discovered that hemorrhagic pituitary adenomas typically demonstrate lower absolute and relative ADC values compared with the other two groups. As a result, the following thresholds of rADC mean > 1.92 or rADC min > 1.07 effectively distinguish craniopharyngiomas, while rADC min > 1.01 effectively separates mucinous RCCs from hemorrhagic pituitary adenomas.

Despite differences in methodology, our results align with previous studies that have reported lower ADC values of hemorrhagic adenomas [14, 15]. Even the optimal threshold of absolute ADC min = 0.7 × 10^−3^mm^2^/s suggested by Mahmoud et al. is not significantly different from our ADC min = 0.82 × 10^−3^mm^2^/s and 0.83 × 10^−3^mm^2^/s, determined for adamantinomatous craniopharyngiomas and mucinous RCCs, respectively. Nevertheless, in routine clinical practice, we recommend the use of the aforementioned relative values, as they appear to be more objective.

In conventional MRI scans, non-functional cystic pituitary adenomas and adamantinomatous craniopharyngiomas often present as a combination of solid and cystic components, making their differential diagnosis challenging. This study demonstrated that distinguishing adamantinomatous craniopharyngiomas can be achieved with ADC mean values > 1.75 × 10^−3^mm^2^/s and rADC mean values > 2.12 (AUC = 0.7). Regarding the study of Kunii et al. on a smaller population, no significant correlations were found [15]. Other studies have compared the ADC values within the solid areas of the lesions in which the results were inconsistent with each other [14, 16]. We claim that, despite achieving a favorable AUC of 0.7 and high specificity of 95% (an overall accuracy of 77%), a sensitivity of 50% may not be sufficient for clinical practice.

In our study, we found that elevated ADC values in every variant of measurement facilitate the diagnosis of serous Rathke’s cleft cysts over non-functional cystic pituitary adenomas. It is important to emphasize that high levels of sensitivity and specificity, mostly around 90%–92%, are result of the measurement method. In our study, serous RCCs were purely cystic lesions, while non-functional cystic pituitary adenomas often contained solid regions. This could lead to reduced ADC mean and min values. Kunii et al. reported no statistically significant difference between the absolute and relative ADC values of the RCCs and the cystic components of pituitary adenomas [15]. However, RCCs were not divided into subgroups based on the various compositions and signal intensity in T1- and T2-weighted sequences, as we did in our study.

Likewise, in our study, serous RCCs demonstrated significantly higher ADC compared to craniopharyngiomas, while mucinous RCCs obtained significantly lower ADC compared to craniopharyngiomas. The first result is consistent with the findings in the publication by Kunii et al. However, the second result contradicts the fact that typically craniopharyngiomas contain higher concentrations of viscous fluids, resulting in lower ADC values. Due to the same concerns about the methodology as above, further investigation is obligatory.

Despite the insufficient number of cases for proper statistical analysis, it is worth mentioning that pituitary abscess and lymphocytic hypophysitis are rare conditions that may present a similar appearance on MRI study [29, 30]. The differentiation can be done based on typically low ADC values observed in sellar abscesses [31]. It has significant clinical importance for patients, as sellar abscess is conventionally managed with early surgical drainage and antibiotic therapy, while lymphocytic hypophysitis is typically treated with high-dose glucocorticoids [32].

This study has several limitations. Firstly, it was initiated on a 1.5 T MR unit, and for the sake of consistency, it had to continue in that setting despite the current preference for higher-field scanners for imaging the sellar and parasellar regions. The need to collect imaging and histopathology data forced us to use the same DWI sequence, although newer, more artifact-resistant DWI sequences have emerged in recent years.

Secondly, the lesions had to be adequately sized, approximately twice the slice thickness, to allow for the measurement of ADC values independently of susceptibility artifacts. Consequently, some lesions were initially excluded from our study.

Finally, the measurement method employed is ROI-based, operator-dependent, and requires an assessment of T1- and T2-weighted images for better pathology evaluation.

In conclusion, DWI provides significant additional value to pituitary MRI examinations. Based on our experience and analysis, the quick scanning time and the additional diagnostic value justify its inclusion into the standard protocol useful in the everyday clinical practice. Moreover, it enhances healthcare cost-effectiveness by preventing the need for supplemental MRI scans and leading to the proper, often conservative treatment method avoiding surgical procedures.

## Data Availability

The magnetic resonance imaging (MRI) data used in this study are available upon reasonable request.
